# RANK expression in EBV positive nasopharyngeal carcinoma metastasis: a ready-to-treat target?

**DOI:** 10.18632/oncotarget.21856

**Published:** 2017-10-16

**Authors:** Carlo Resteghini, Salvatore Alfieri, Pasquale Quattrone, Francesca Dominoni, Giovanna Garzone, Ester Orlandi, Laura Locati, Cristiana Bergamini, Donata Galbiati, Nicola Alessandro Iacovelli, Carlo Fallai, Lisa Licitra, Paolo Bossi

**Affiliations:** ^1^ Head and Neck Medical Oncology Unit, Fondazione IRCCS Istituto Nazionale dei Tumori, 20133 Milan, Italy; ^2^ Department of Pathology and Laboratory Medicine, Fondazione IRCCS Istituto Nazionale dei Tumori, 20133 Milan, Italy; ^3^ Radiation Oncology Unit 2, Fondazione IRCCS Istituto Nazionale dei Tumori, 20133 Milan, Italy

**Keywords:** RANK, nasopharyngeal carcinoma, Epstein Barr virus, Tregs, denosumab

## Abstract

Epstein Barr Virus (EBV) related Nasopharyngeal Carcinoma (NPC), is an highly chemo- and radiosensitive endemic malignancy in southeast Asia. More than one third of locally advanced cases relapse after curative treatment, especially because of bone, liver and lung metastases. Lymphocyte sub-populations favour EBV-associated carcinogenesis and tumour progression and several strategies aim to reverse this phenomenon. Receptor activator of NF-kB (RANK) and its Ligand (RANKL), key regulator of bone metabolisms, are expressed in several malignancies and tumor-infiltrating Tregs. We collected 17 paired FFPE specimen of primary and metachronous metastatic or regionally relapsed EBV related NPC and evaluated RANK expression by immunohistochemistry. All primary tumour specimens resulted not evaluable whereas all metastatic specimens, regardless of sites, showed high RANK IHC expression in the tumor with no staining in normal surrounding tissues. This observation deserves further clarifications and could open the way to trials testing the hypotesis that targeting the RANK/RANKL pathway with denosumab, an already available, clinically approved monoclonal antibody for metastatic bone lesions, might restore proper anti-tumor immune response in NPC metastatic patients.

## INTRODUCTION

Epstein Barr Virus (EBV) related Nasopharyngeal Carcinoma (NPC), while rare in most of the world, is an endemic malignancy in southeast Asia [1]. Despite being highly chemo- and radiosensitive [2], more than one third of locally advanced cases relapse after curative treatment, especially because of bone, liver and lung metastases [3, 4].

Althought NPC express viral antigens (e.g. LMP1 and EBNA) [5], as in many other virus related tumors the immune system does not play an active role contrasting tumor growth and spread. For instance, there is evidence that lymphocyte sub-populations favour EBV-associated carcinogenesis and tumour progression [6]. Several strategies aim to reverse this phenomenon, such as restoring CD8+ T cell response via Tregs depletion to recreate an effective antitumor immunologic environment [7] or EBV-targeted cell therapy with autologous virus-specific cytotoxic T lymphocytes [8].

Receptor activator of NF-kB (RANK) and its Ligand (RANKL) are key regulators of bone metabolisms, especially in the metastatic nice, where they interact with the immune microenvironment [9–11]. In several malignancies, such as breast, prostate cancer and osteosarcoma, RANK/RANKL expression has been found on both primary and metastatic cells [12–15], as well as in tumor-infiltrating Tregs, known for inhibiting antitumoral immunity [16–17]. Furthermore, in a non-cancer context, it has been reported that RANKL and RANK signals are implicated in Treg cell expansion [18, 19].

All these premeses lead to the hypothesis that targeting the RANK/RANKL pathway with denosumab, an already available, clinically approved monoclonal antibody for metastatic bone lesions, might restore proper anti-tumor immune response in NPC metastatic patients. In order to provide data supporting this hypothesis, we evaluated whether EBV related NPC primary and metastatic tissue present RANK expression.

## RESULTS

**Figure 1:**
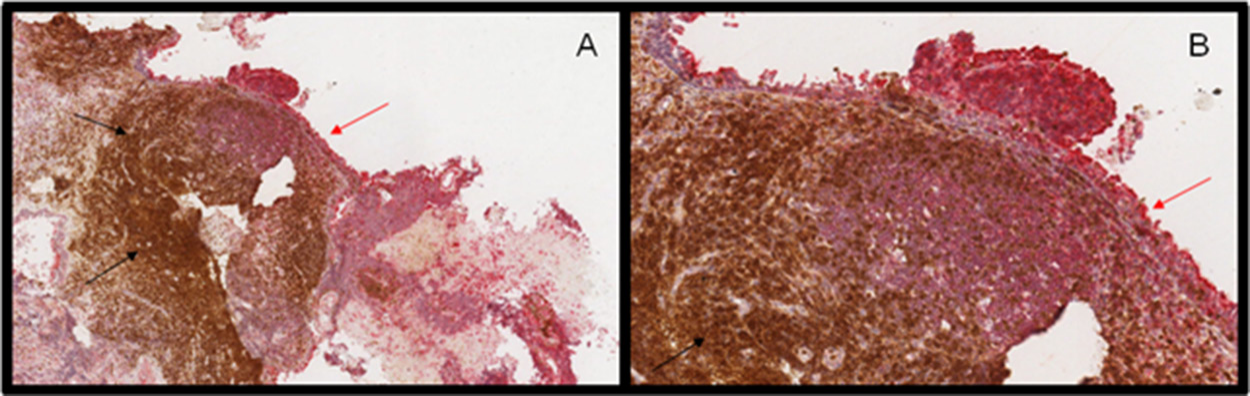
**(A-B)** RANK (red arrows) and CD3/CD20 (black arrows) double staining of primary nasopharyngheal carcionoma. RANK expression is evident in normal nasopharyngeal cells while tumor cells, surrounded by lymphoid infiltrate, present with weak stining.

Primary lesions analysed with RANK IHC showed strong background staining due to the lymphatic infiltrate present in the specimens that prevented the differential evaluation of the proper tumor staining. In order to evidentiate the lymphatic infiltrate, double staining for pooled CD3/CD20 and RANK was performed on NPC primary and metastatic lesion. Furthermore, we performed the same double staining on primary and nodal breast cancer lesion to provide a positive control. Nevertheless, aspecific RANK staining characterized the NPC spacimens (Figure 1), with uncertian resultes despite several attempts with different technical approach.

**Figure 2:**
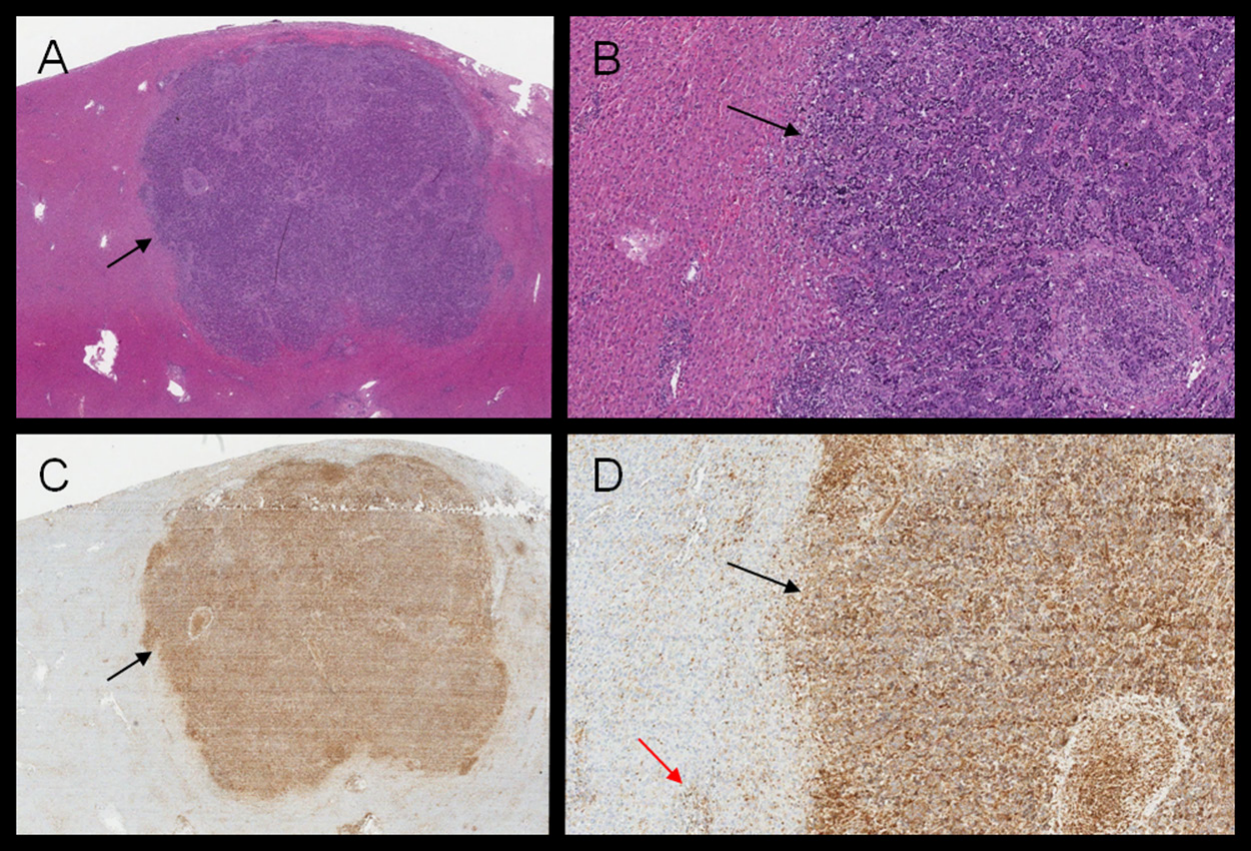
**(A-B)** H&E of liver biopsy showing NPC localization. **(C-D)** RANK IHC; positive areas of RANK expression overlaps with NPC cells (black arrows), while Kupfer cells (red arrow) in normal liver tissue represents positive internal control.

**Figure 3:**
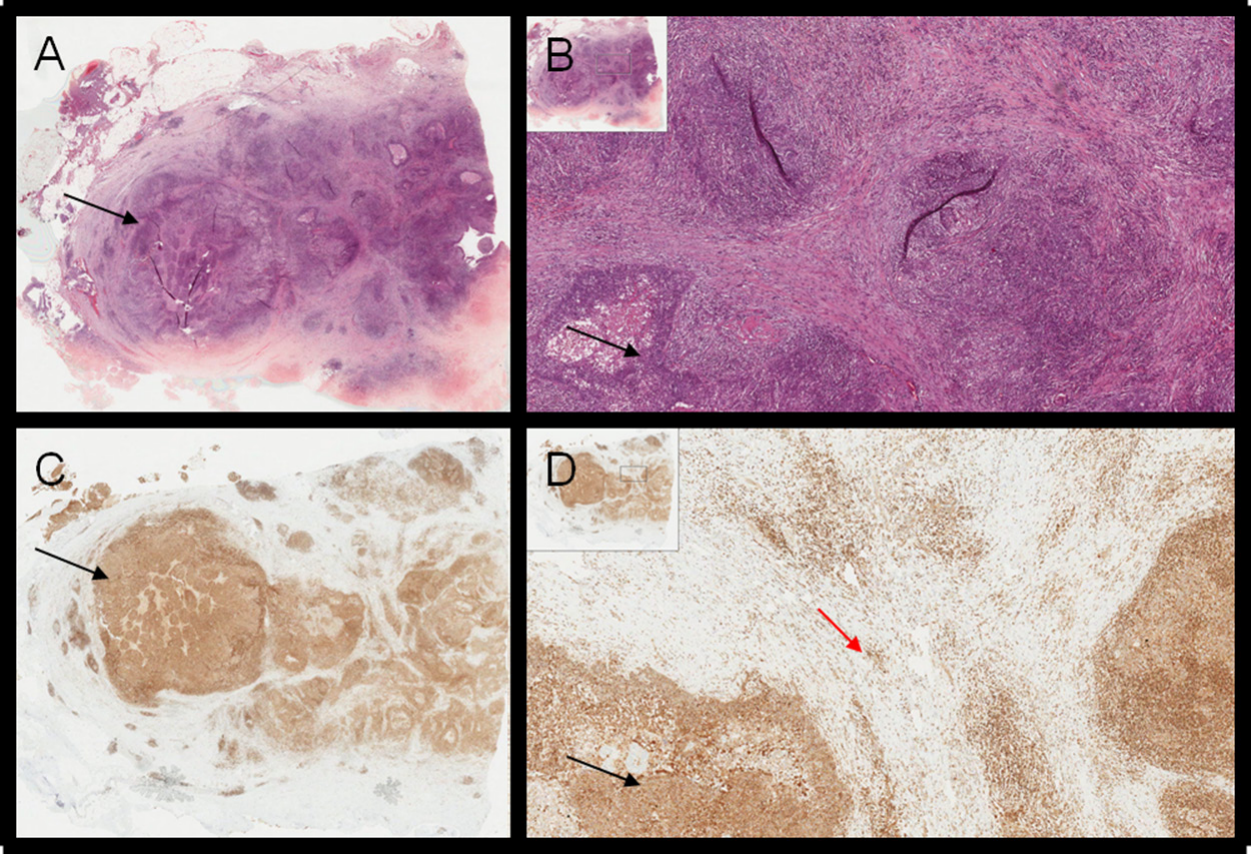
**(A-B)** H&E of lymph node metastasis of NPC; **(C-D)** corresponding RANK staining (black arrows); white cells provide the positive internal control in the context of neoplastic island (red arrow).

**Figure 4:**
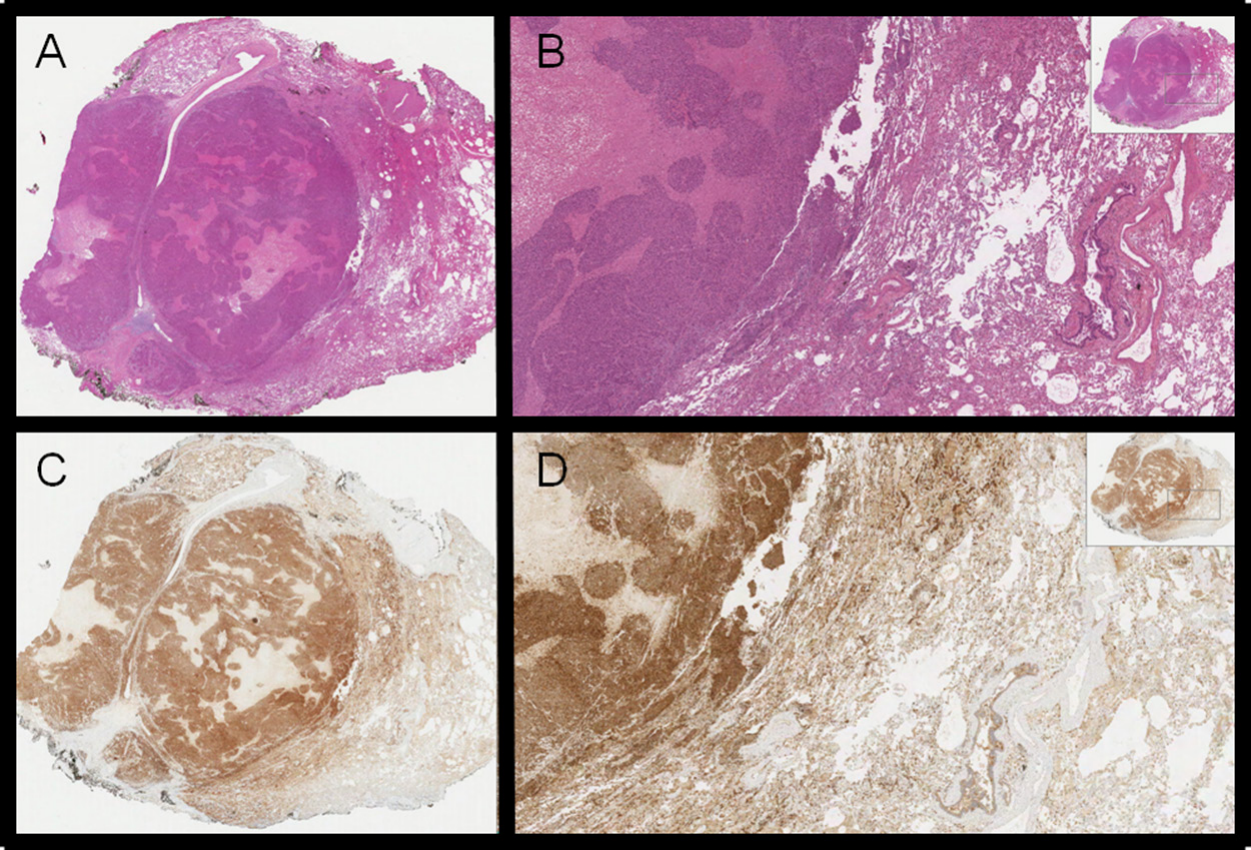
H&E **(A-B)** and RANK positive staining **(C-D)** of NPC lung metastasis.

Specimens from nodal or metastatic cases had the following distributions: neck or metastatic nodes 7/19 (37%); liver 5/19 (26%); lung 3/19 (16%); bone 2/19 (10,5%); soft tissue 2/19 (10,5%) (Table 1). Two cases resulted not evaluable due to tissue necrosis or strong background staining from nodes lymphocytes. All other metastatic specimens, regardless of sites, showed high RANK IHC expression in the tumor with no staining in normal surrounding tissues (Figures 2–4).

**Table 1:** Spacimens distribution and staining results

Case #	REC/MTS	Tissue Analysed	Rank IHC
1	REC	NODE	POS
2	REC	NODE	POS
3	REC	NODE	POS
4	REC	NODE	NOT EVALUABLE^*^
5	MTS	NODE	NOT EVALUABLE ^**^
6	MTS	NODE	POS
7	REC	NODE	POS
8	REC	LIVER	POS
9	MTS	LIVER	POS
10	MTS	LIVER	POS
11	MTS	LIVER	POS
12	MTS	LIVER	POS
13	REC	NECK SOFT TISSUE	POS
14	REC	NECK SOFT TISSUE	POS
15	MTS	LUNG	POS
16	MTS	LUNG	POS
17	MTS	LUNG	POS

^*^ too much background staining from lymphoid infiltrate; ^**^ necrosis.

## DISCUSSION

We documented RANK expression by NPC metastatic tissue via IHC. This observation deserves further clarifications. An area worth investigation would be the possibility to determine RANK expression in primary lesion of localised disease at presentation and the correlation with prognosis. Indeed, this evaluatoin might lead to the identification of RANK as a possible marker of more aggressive disease, highlighting the implication of RANK pathway in disease progression and the metastatic process. Primary lesion evaluation could also beter select a population with locoregionally advanced neoplasia who would benefit from adjuvant treatment.

We encountered difficulties in RANK determination on primary lesion, and this represents a limitation of the present study. We addressed this issue by performing a double staining to differentiate the tumor and the lymphoid infiltrate, with equivocal results.

Although the present study does not throughtly explore the relationship between the immune context and RANK expression by nasopharyngeal carcinoma, we consider safe to assume that the already known links between RANK/RANKL pathway and Treg in neoplastic context can be extended also to nasopharyngeal pathology, given the established role of Treg in NPC tumorogenesis and progression [24–26] and our newly highlighted connection between NPC and RANK.

Our results are consistent with other observation in different pathology. Indeed, breast and prostate cancer, as well as osteosarcoma, has shown RANK/RANKL expression on both primary and metastatic cells [2, 3]. Furthermore, our results appears to be solid, even on a small sample size, given the 100% positive RANK staining on evaluable samples.

Lastly, the greatest implication of our observation is the availability of a safe and already approved drug to target RANK/RANKL pathway, with potential clinical benefit behind bone tournover. Our early results could open the way to trials aimed at reinforcing immunological response against the tumor through the blockage of Tregs regulated by RANKL, providing a clinical benefit as reported in other diseases [20–23]. In light of the data recently published in melanoma regarding the potential synergistic effect of anti-CTLA4 and anti-RANKL drugs described in preclinical model [24], our observation could open the possibility for a clinical trial invastigatig the combination of immunotherapeutic agents plus anti-RANKL drug also in metastatic nasopharyngeal cancer. Early clinical data have already shown that checkpoint inhibition like pembrolizumab provide benefits in a subset of metastatic NPC. Therefore, combining more immune-modulating agents might prove to be a useful, novel strategies.

In conclusion, we consider that these simple but thrustable observations could offer a strategy for unlocking anti tumor immunity, providing a possible new treatment opportunity in virally-driven NPC patients.

## MATERIALS AND METHODS

We conducted a retrospective analysis collecting 17 paired FFPE specimen of primary and metachronous metastatic or regionally relapsed EBV-related NPC treated in our Institution.

Rank was identified through samples fixed in 4% neutral buffered formaldehyde. Representative tumor blocks were sectioned at 3μ thickness. IHC was performed by the EnVision FLEX+, mouse, hight pH (Link) method (Dako Denmark A/S Produktionsvej 42 DK-2600 Glostrup) with Dako Autostainer Link48. A mouse monoclonal antibody against RANK protein (clone 80707, R&D Systems, Inc., Minneapolis, MN) was used at a concentration of 25 μg/ml. 3-3’-Diaminobenzidine (DAB) was used for color development and Mayer's hematoxylin was used for counterstaining.

In order to address the differential evaluation of RANK staining in primary lesions and lymphatic infiltrate, we selected two NPC cases as well as two breast cancer cases as positive control. On these saples, double staining for pooled CD3/CD20 and RANK was performed on NPC and breast cancer tissue to evidentiate the lymphoid infiltrate (CD3/CD20 Dako Denmark A/S Produktionsvej 42 DK-2600 Glostrup). 3-3’-Diaminobenzidine (DAB) was used for CD3/CD20 color development, warp red Chromogen Kit for RANK stainig and Mayer's hematoxylin was used for counterstaining.

## Abbreviations

RANK: Receptor activator of NF-kB
RANKL: Receptor activator of NF-kB Ligand
EBV: Epstein Barr Virus
NPC: Nasopharyngeal carcinoma
